# Establishment and Characterization of 5-Fluorouracil-Resistant Human Colorectal Cancer Stem-Like Cells: Tumor Dynamics under Selection Pressure

**DOI:** 10.3390/ijms20081817

**Published:** 2019-04-12

**Authors:** Maria Giovanna Francipane, Denis Bulanin, Eric Lagasse

**Affiliations:** 1 McGowan Institute for Regenerative Medicine and Department of Pathology, University of Pittsburgh School of Medicine, Pittsburgh, PA 15219, USA; lagasse@pitt.edu; 2 Ri.MED Foundation, 90133 Palermo, Italy; 3 Department of Biomedical Sciences, Nazarbayev University School of Medicine, Astana 010000, Kazakhstan; dbulanin@nu.edu.kz

**Keywords:** colorectal cancer, chemoresistance, cancer stem cells

## Abstract

5-Fluorouracil (5-FU) remains the gold standard of first-line treatment for colorectal cancer (CRC). Although it may initially debulk the tumor mass, relapses frequently occur, indicating the existence of cancer cells that are therapy-resistant and are capable of refueling tumor growth. To identify mechanisms of drug resistance, CRC stem-like cells were subjected to long-term 5-FU selection using either intermittent treatment regimen with the IC50 drug dose or continuous treatment regimen with escalating drug doses. Parental cancer cells were cultivated in parallel. Real-time PCR arrays and bioinformatic tools were used to investigate gene expression changes. We found the first method selected for cancer cells with more aggressive features. We therefore transplanted these cancer cells or parental cells in mice, and again, found that not only did the 5-FU-selected cancer cells generate more aggressive tumors with respect to their parental counterpart, but they also showed a different gene expression pattern as compared to what we had observed in vitro, with *ID1* the top upregulated gene. We propose ID1 as a stemness marker pervasively expressed in secondary lesions emerging after completion of chemotherapy.

## 1. Introduction

Current first-line chemotherapy generally consists of cytotoxic agents, like 5-fluorouracil (5-FU), a uracil analogue that following intracellular conversion into three active metabolites (fluorouridine triphosphate, fluorodeoxyuridine triphosphate, and fluorodeoxyuridine monophosphate) causes alterations in RNA processing/function and severe DNA damage [1]. Evidence shows that DNA lesions are able to trigger apoptosis [2]. However, evasion of both death receptor and mitochondrial pathways is common in response to stress stimuli [3]. Antiapoptotic mechanisms together with cytoprotective autophagy [4], upregulated DNA repair pathways [5], and metabolic rewiring [6] allow tumor cells to thrive in conditions that otherwise would be lethal propagating the disease.

Despite intense studies, one important question still remains open: are resistant cells already present in the tumor at the time of first treatment or do tumor cells adapt to therapy? Several models have been proposed to address this important question. The longstanding cancer model, known as ‘clonal evolution’ postulates that tumors arise from a single mutated cell. This cell, in turn, generates an abnormal offspring that also mutates, forming a mass of genetically diverse tumor cells [7]. From this perspective, tumor progression proceeds via a process analogous to Darwinian evolution, where only cells with advantageous mutations survive and fuel tumor growth [8]. The opposing cancer stem cell (CSC) theory proposes instead that tumor growth is driven and sustained by a rare population of cells capable of self-renewal and differentiation [9,10]. While the CSC theory could explain the hierarchical heterogeneity observed in most tumors, and why some patients suffer relapses after initial successful therapy [11], clonal evolution driven by genomic instability is likely to play an important role in tumor progression by allowing CSCs to adapt to environmental, immunologic and pharmacologic cues [12]. Indeed, tumors are dynamic entities and understanding how their cells succeed in offsetting survival pressures imposed by therapy is of utmost importance for the development of more effective treatments.

Current drug design has been largely focused on initial efficacy, and the effects of drug selective pressures remain poorly studied. Panels of chemotherapy resistant cancer cell lines have been generated to investigate the molecular pathways that may be associated with resistance. However, the vast majority of these studies has been performed on commercially available cell lines and not on primary cells isolated from patients’ biopsies. Both pulse and incremental drug treatment methods have been described. However, a comparison of gene expression in the respective treatment-derivatives remains incomplete. Moreover, to our knowledge, re-transplantation into an in vivo setting of such in vitro established resistant cell lines has been poorly addressed.

In this manuscript, we used stem cells that were identified, isolated, and characterized in our laboratory from colorectal cancer (CRC) patients’ specimens [13]. We generated 5-FU resistant cells by using either intermittent treatment with the IC50 drug dose or continuous treatment with escalating drug doses. We then used real-time PCR arrays and ingenuity pathway analysis (IPA) to investigate gene expression changes. Our results indicated that the intermittent treatment with the IC50 of 5-FU could serve as a better model to investigate drug resistance. While biological functions like cell survival, proliferation, movement, invasion, and migration were predicted by IPA to be inhibited in cells undergoing continuous treatment with escalating 5-FU doses with respect to parental cells passaged in parallel, the same functions were predicted to be unaffected in cells undergoing intermittent treatment with the IC50 5-FU dose. Moreover, while 5-FU resistant cells under continuous treatment with escalating drug doses were predicted to be more prone to cell death with respect to parental cells, cell cycle progression was not predicted to be delayed in the other cell group despite DNA damage, suggesting a propensity to genomic instability, as previously described [13]. Tumor xenografts were generated from both parental and resistant cells undergoing intermittent treatment with the IC50 5-FU dose with the resistant cells generating more aggressive phenotypes. The gene expression changes observed in the in vivo xenograft setting were compared to those observed in the in vitro setting. We found in vivo and in vitro gene expression patterns to differ, indicating a capability for 5-FU resistant cells to dynamically adapt and evolve. We identified *ID1* as the top upregulated gene in the tumor generated by 5-FU resistant cells.

## 2. Results

### 2.1. Establishment of 5-FU Resistant Human CRC Stem-Like Cells

**Figure 1:**
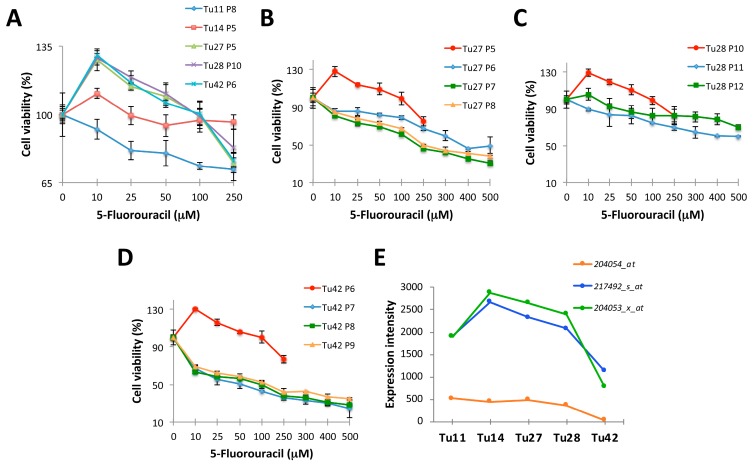
Establishment of 5-FU resistant human colorectal cancer (CRC) stem-like cells. (**A**) Line graphs showing cell viability (%) of Tu11, Tu14, Tu27, Tu28, and Tu42 CRC stem-like cells (P stands for passage) treated with vehicle or serial dilutions (10–250 µM) of 5-FU. Data are expressed as mean percentage (± SD) of cell numbers relative to control culture. (**B**–**D**) Line graphs showing cell viability (%) of consecutive passages of Tu27, Tu28, and Tu42 CRC stem-like cells treated with vehicle or serial dilutions (10–500 µM) of 5-FU. Data are expressed as mean percentage (± SD) of cell numbers relative to control culture. (**E**) Line graph showing the expression intensity of *PTEN*/*MMAC1*/*TEP1* in short-term feeder-expanded Tu11, Tu14, Tu27, Tu28, and Tu42 CRC stem cells obtained using three different microarray probes (204054_at; 217492_s_at; 204053_x_at).

CRC stem cells were derived from five human metastatic cancers (Tu11, Tu14, Tu27, Tu28, and Tu42) using a feeder-dependent cell culture system, previously described by our laboratory [13]. In order to establish chemotherapeutic resistant cells, we first tested the ability of tumor cells to grow in the absence of the feeder layer. Complete depletion of the feeder cells was obtained at the second passage of tumor cells on plastic (data not shown). At this passage, tumor cells often showed a high ratio of nucleus to cytoplasm, prominent nucleoli, and colony morphology similar to that of embryonic stem cells (data not shown). However, following repeated passages, colonies with these characteristics became less frequent. These passaged feeder-free cells will be hereinafter referred to as ‘stem-like cells’. After one passage on the feeder layer, and two passages on plastic, all five cell lines were treated with serial dilutions of 5-FU to establish an IC50 dose. Low 5-FU doses (10-25 µM) surprisingly led to increased cell numbers in most of the cultures (Figure 1A).

We found the subpopulation of cells expressing the cancer-initiating cell marker EpCAM [14] to proliferate in response to low 5-FU doses. Indeed, a higher number of cells stained positive for EpCAM after treatment, and only EpCAM positive cells also stained positive for the proliferation cell marker Ki-67 (data not shown). Unfortunately, we were not able to further propagate Tu11 and Tu14 cells. In addition, Tu28 cells survived for only two more passages on plastic, while Tu27 and Tu42 cells were easily expanded (Figure 1B–D). Along with passages, these cells became less resistant to 5-FU (IC50 for Tu27 = 250 µM; IC50 for Tu42 = 100 µM) (Figure 1B,D).

Once the IC50 dose for 5-FU was established, we attempted the generation of 5-FU resistant cells by either intermittent treatment with the IC50 drug dose (hereinafter called R1 cells; two pulses of 100 µM 5-FU were given) or continuous treatment with escalating drug doses (hereinafter called R2 cells; six doses were given, starting with the 1/20 IC50 dose up to the IC50 dose). Unfortunately, we were not able to establish stable 5-FU resistant Tu27 cells: as early as 5 weeks after the start of treatment, all cells died. Conversely, we successfully generated resistant Tu42 cells.

We retrospectively reviewed microarray data from short-term feeder-expanded Tu11, Tu14, Tu27, Tu28, and Tu42 CRC stem cells to investigate why we failed to generate resistant cancer cell lines most of the time. We found that with respect to the other cell lines, Tu42 cells downregulated the tumor suppressor *PTEN*/*MMAC1*/*TEP1* (PTEN, phosphatase deleted on chromosome ten; MMAC1, mutated in multiple advanced cancers; TEP1, tensin-like phosphatase) (Figure 1E). Thus, *PTEN*/*MMAC1*/*TEP1* status might be a crucial determinant of 5-FU resistance in CRC.

**Figure 2:**
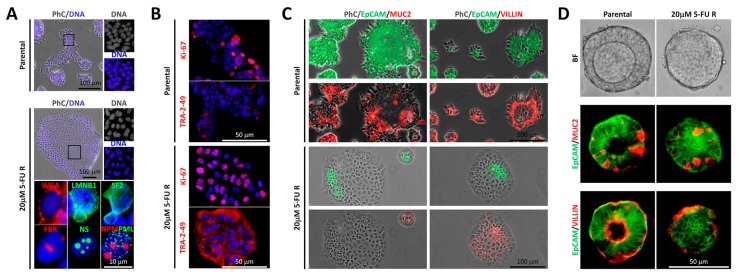
Features of the human colorectal cancer (CRC) stem-like cell line Tu42 under low 5-FU doses. (**A**) Merged images of phase contrast (PhC) and Hoechst-stained nuclei (DNA) of parental (top) or 20 µM 5-FU resistant (center) Tu42 cells. Boxed areas are shown on the right at higher power and indicate the presence of distinct large foci in response to low-dose 5-FU. Immunofluorescence staining for Wheat germ agglutinin (WGA), Laminin B1 (LMNB1), SF2, Fibrillarin (FBR), Nucleostemin (NS), Nucleophosmin (NPM), or PML in 20 µM 5-FU resistant Tu42 cells (bottom) suggested high nucleus-to-cytoplasm ratio, the presence of nuclear buds, and identified the previously observed foci as nucleolar structures. Nuclei were counterstained using Hoechst. Pictures are representative of three independent experiments. (**B**) Immunofluorescence staining for Ki-67 or TRA-2-49 of parental (top) or 20 µM 5-FU resistant (bottom) Tu42 cells indicated the accumulation of Ki-67 in nucleolar regions of resistant cells and the upregulation of TRA-2-49 in the same cells with respect to parental cells. Nuclei were counterstained using Hoechst. Pictures are representative of three independent experiments. (**C**) Merged images of phase contrast (PhC), EpCAM, and Mucin2 (Muc2), or PhC, EpCAM, and Villin of parental (top) or 20 µM 5-FU resistant (bottom) Tu42 cells indicated that resistant cells downregulate EpCAM, Muc2, and Villin, as compared to parental cells. Pictures are representative of three independent experiments. (**D**) Bright field (BF) images of live unstained parental (top left) or 20 µM 5-FU resistant (top right) Tu42 cells forming organoids in Matrigel, and immunofluorescence staining for EpCAM/Muc2 (center) or EpCAM/Villin (bottom) in their respective frozen sections indicated resistant cells reacquire EpCAM expression in three-dimensional (3D) cell culture but fail to generate organoids with a central lumen. Pictures are representative of three independent experiments.

Low 5-FU doses (up to the 20 µM) resulted in accumulation of large foci in Tu42 cells (Figure 2A, middle panels).

Immunofluorescence staining for Wheat germ agglutinin (WGA), Laminin B1 (LMNB1), SF2, Fibrillarin (FBR), Nucleostemin (NS), Nucleophosmin (NPM), or PML suggested high nucleus-to-cytoplasm ratio in these cells, as well as the presence of nuclear buds, and identified the previously observed foci as nucleolar structures (Figure 2A, bottom panels). Interestingly, the nucleolar structures were highly reactive for Ki-67 (Figure 2B), indicating that the cells were likely in interphase and responding to DNA damage [15,16]. Moreover, with respect to parental cells, 20 µM 5FU-resistant cells upregulated the pluripotent stem cell marker TRA-2-49 [17] (Figure 2B) and downregulated EpCAM, Mucin 2 (Muc2, the major secretory mucin synthesized and secreted by goblet cells [18]), and Villin (a cytoskeletal protein and a differentiation marker [19]) (Figure 2C). Interestingly, resistant cells could reacquire EpCAM expression when directed to form organoids in Matrigel, although they failed to generate organoids with a central lumen (Figure 2D). These observations suggest two possible outcomes: a) low 5-FU doses given under two-dimensional culture conditions reprogram CRC stem-like cells into less differentiated CSCs and this reprogramming is partially reversible when the cells are cultured in three-dimensional (3D) matrices; or b) the selective pressure under low 5-FU doses allows survival of less differentiated CSCs already present in the culture.

### 2.2. Differential Morphology, Gene Expression Profiles, and Biological Functions in 5-FU Resistant Tu42 CRC Stem-Like Cells as Compared to Parental Cells

**Figure 3:**
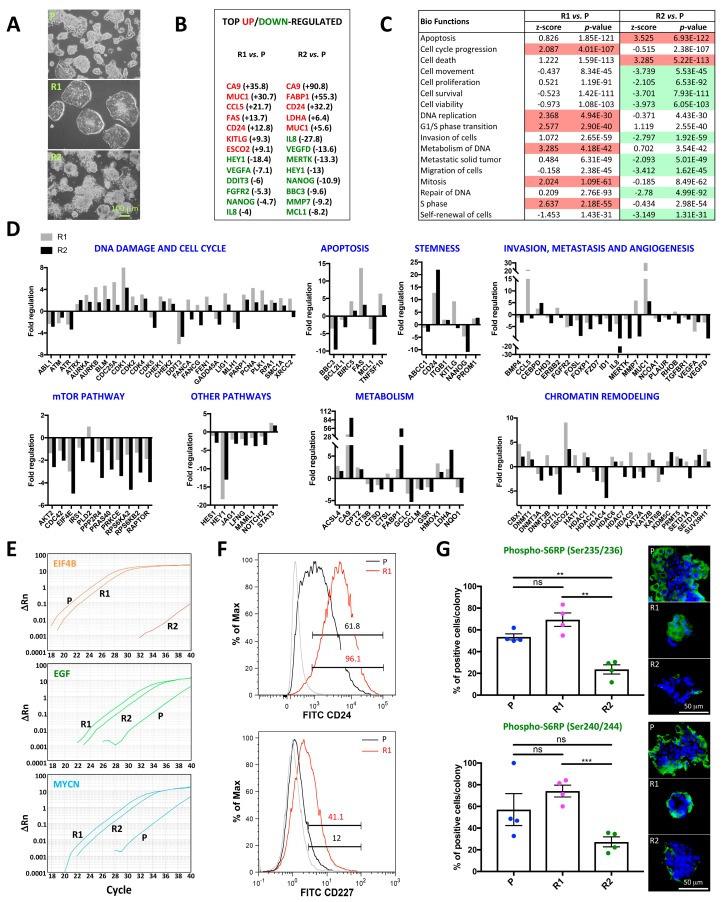
Differential morphology, gene expression profiles and biological functions in 5-FU resistant Tu42 colorectal cancer (CRC) stem-like cells as compared to parental cells. (**A**) Phase contrast images of parental (P) or 5-FU resistant Tu42 cells selected over an 8-month period by either intermittent treatment with the IC50 drug dose (R1; two pulses of 100 µM 5-FU were given) or continuous treatment with escalating drug doses (R2; six doses were given, starting with the 1/20 IC50 dose up to the IC50 dose). (**B**) List of the top up- or downregulated genes in 5-FU resistant Tu42 cells as compared to parental cells obtained by real-time PCR arrays. (**C**) Biological functions arranged in alphabetical order predicted by IPA to be activated (red) or inhibited (green) in 5-FU resistant Tu42 cells as compared to parental cells. (**D**) Bar graphs show fold regulation (positive/negative fold changes calculated from two technical replicates) of genes involved in DNA damage and cell cycle, apoptosis, stemness, invasion, metastasis and angiogenesis, mTOR pathway, other pathways, metabolism, and chromatin remodeling in 5-FU resistant Tu42 cells as compared to parental cells (grey bars: R1 cells versus parental cells; black bars: R2 cells versus parental cells). (**E**) Representative amplification plots of *EIF4B*, *EGF*, and *MYCN* in parental and 5-FU resistant Tu42 cells. ΔRn is plotted against PCR cycle number. (**F**) Flow cytometry histograms showing expression of CD24 and CD227 (Muc1) in parental (black histograms) and R1 (red histograms) cells (grey histograms indicate unstained control). Histograms are representative of three independent experiments. (**G**) Scatter dot plot with mean and SEM showing percentages of phospho-S6RP (pS6RP-S235/236 and pS6RP-S240/244) positive cells/colony generated by parental, R1, or R2 cells. Each dot represents a colony. Statistical significance, calculated using unpaired *t*-test with Welch’s correction, is shown (ns, *p* > 0.05; **, *p* ≤ 0.01; ***, *p* ≤ 0.001). Representative pS6RP-S235/236 and pS6RP-S240/244 stained colonies are shown at the right of each graph. Data are representative of three independent experiments.

While parental cells passaged in parallel progressively acquired a differentiated morphology comprising crypt–villus structures (Figure 3A, top image), R1 cells organized in round colonies with defined edges following 8 months of 5-FU-induced selection pressure (Figure 3A, middle image). Conversely, R2 cells treated with increasing drug doses (up to the 20 µM 5-FU dose) were initially organized in round colonies (data not shown) and eventually showed a more differentiated morphology that was similar to the parental cells (Figure 3A, bottom image).

We isolated RNA from all three-cell groups (parental, R1, and R2) derived from Tu42, and analyzed 588 genes using several RT^2^ Profiler PCR arrays in 96-well format. Each 96-well plate included 84 pathway- or disease-focused genes, as well as five housekeeping genes. Moreover, each plate included control elements for genomic DNA contamination detection, RNA sample quality, and three replicate controls to check general PCR performance. We found the system to be very reliable and reproducible, with Pearson correlation of gene expression between two technical replicates >0.99 (Supplementary Figure S1). Figure 3B shows the list of top up/downregulated genes in R1 and R2 cells with respect to parental cells. Both resistant cell groups upregulated *CA9*, *MUC1*, and *CD24*, while they downregulated *HEY1*, *NANOG*, and *IL8*. Biological functions including cell cycle progression, DNA replication, G1/S phase transition, metabolism of DNA, mitosis, and S phase were predicted by IPA to be activated in R1 cells with respect to parental cells (Figure 3C). Conversely, biological functions including cell movement, cell proliferation, cell survival, cell viability, invasion of cells, metastatic solid tumor, migration of cells, repair of DNA, and self-renewal of cells were predicted to be inhibited in R2 cells with respect to parental cells (Figure 3C). R2 cells were also predicted to activate biological functions like apoptosis and cell death (Figure 3C). Thus, the intermittent treatment with the IC50 drug dose selected for cells with more aggressive features than the continuous treatment with escalating drug doses. Genes expressed differentially between parental and R1 cells or parental and R2 cells are shown in Figure 3D. Official full names for each reported gene are listed in Table 1.

#### 2.2.1. DNA Damage and Cell Cycle

Whereas normal cells or drug-responsive cancer cells repair DNA damage and go into senescence or apoptosis, loss of cell cycle checkpoints allows cancer cells to proliferate and perpetuate harmful mutations [10]. We analyzed multiple DNA damage and cell cycle-related genes in resistant cell derivatives. The top upregulated genes of these categories in R1 cells included *CDK1*, *CDC25A*, *BLM*, and *AURKB*. CDK1 and CDC25A force proliferation despite drug-induced DNA damage, due to their ability to completely disable cell-cycle checkpoints [20,21], and are therefore associated with tumor progression and drug resistance [22]. The *BLM* product is a helicase of the RECQ family with roles in DNA replication and repair [23]. AURKB overexpression is associated with multinucleation and polyploidy and therefore has a high correlation with genomic instability [24]. *CDK1* and *BLM* were also upregulated in R2 cells, although to a lower extent. Finally, both R1 and R2 cells downregulated *DDIT3*, which has been implicated in stress responses leading to apoptosis [25].

#### 2.2.2. Apoptosis

DNA-damaging agents induce apoptosis through both the mitochondrial and the death receptor pathway. Surprisingly, both R1 and R2 cells upregulated *FAS* and *TNFSF10*. While the overwhelming majority of studies on FAS and TNFSF10 have explored their role as initiators of apoptosis, sporadic reports suggest that they could actually be drivers of cell proliferation, migration, and invasion, and therefore contributors of tumor progression [26]. The tumorigenic activity of FAS is likely mediated by a pathway involving JNK and c-Jun. [27]. On the other hand, TNFSF10 can antagonize apoptosis induction and enhance proliferation through activation of the NF-κB pathway [28]. R1 cells also upregulated *BIRC5*. Mostly known as an anti-apoptotic factor, BIRC5 additionally controls cell division by regulating the G2/M phase [29]. The expression levels of the anti-apoptotic genes *BBC3* and *MCL1* were reduced in both resistant cell groups, especially in R2 cells. Moreover, R2 cells also downregulated an additional anti-apoptotic gene, *BCL2L1*.

#### 2.2.3. Stemness

Both R1 and R2 cells upregulated the putative CSC marker *CD24* and *PROM1*, to a lower extent. Both markers correlate with invasiveness in CRC [30]. *KITLG*, encoding the ligand for the c-kit proto-oncogene [31], was highly upregulated in R1 cells, but not in R2 cells. Both cell groups downregulated the pluripotent stem cell marker *NANOG* [32], especially R2 cells. Finally, *ABCC1*, which encodes a multidrug resistance-associated protein [33], was downregulated in R2 cells but not in R1 cells.

#### 2.2.4. Invasion, Metastasis, and Angiogenesis

R1 cells showed strong upregulation of three genes positively associated with migration, invasion and metastasis: *CCL5* [34], *MUC1* [35], and *MMP7* [36]. While *MUC1* was also upregulated in R2 cells (although to a much lower extent than in R1 cells), *CCL5* was unchanged and *MMP7* was highly downregulated in R2 cells. Another gene, associated with cell invasiveness, *CHD3* [37], was downregulated in R2 cells while unchanged in R1 cells. Genes encoding proteins with oncogenic characteristics including *MERTK* [38], *ID1* [39], *FZD7* [40], *NCOA1* [41], *PLAUR* [42], and *TGFBR1* [43] were downregulated in R2 cells while unchanged or slightly changed in R1 cells. Another gene encoding an oncogene, *ERBB2* [44], was upregulated in R1 cells while unchanged in R2 cells. Genes with a tumor suppressive role including *FOXP1* [45] and *FGFR2* [46] were downregulated in both cell groups. Pro-angiogenetic genes, including *VEGFD* [47], *FOSL* [48], and *IL8* [49] were decreased in both cell groups, especially in R2 cells. Another pro-angiogenetic gene, *VEGFA* [47], was also reduced in both cell groups, but its levels were lower in R1 cells.

#### 2.2.5. mTOR Pathway and Other Pathways

Several genes implicated in the mTOR pathway [50,51] were downregulated in R2 cells and unchanged or slightly changed in R1 cells. With the exception of *HEY1*, several genes encoding members of the Notch family (*JAG1*, *LFNG*, *MAML1*, *NOTCH2* [52]) showed a more enhanced downregulation in R2 cells than R1 cells. Notch pathway activation has been suggested to function as a protective mechanism in response to chemotherapy [53]. Finally, *STAT3* was slightly upregulated in R1 cells.

#### 2.2.6. Metabolism

Metabolic alterations are common in tumors. These include increased uptake of glucose, amino acids, and lipids. Under normal physiological conditions, glucose is catabolized to pyruvate. Most of the pyruvate undergoes several changes to become acetyl-CoA and enter the Kreb’s cycle. Only a small amount of pyruvate is converted to alanine or lactate. In contrast, tumor cells prefer lactic acid fermentation as a source of energy; that is, they convert pyruvate to lactate even under normoxic conditions. This phenomenon, referred to as the Warburg effect [54], is possible thanks to the upregulation of LDHA, a key glycolytic enzyme [55]. We found *LDHA* to be upregulated in both cell groups, especially in R2 cells. Carbon dioxide is released during aerobic glycolysis, leading to a decrease in the intracellular and extracellular pH. Studies have shown that cancer cells adapt to this acidic milieu thanks to the activity of carbonic anhydrases including CA9, which catalyzes the conversion of carbon dioxide into bicarbonate [56]. *CA9* was highly upregulated in both groups, especially in R2 cells. Lipid metabolism is often elevated in cancer cells and plays an important role in their growth and malignancy. *FABP1*, which encodes a protein critical for fatty acid uptake and intracellular transport [57] was upregulated in both cell groups, especially in R2 cells. Other genes whose products are lysosomal proteases involved in lipid metabolism (*CTSB*, *CTSD*, and *CTSL* [58]) were also downregulated in R2 cells. Finally, resistant cells also showed alterations of genes encoding proteins involved in fatty acid synthesis (*ACSL4* [59]) or oxidation (*CPT2* [60]), as well as glutathione metabolism (*GCLC*, *GCLM*, *GSR*, and *NQO1* [61]) and oxidative stress (*HMOX1* [62]).

#### 2.2.7. Chromatin Remodeling

The chromatin remodeling genes which showed the greatest changes in R1 cells were *ESCO2*, *CBX1*, and *SUV39H1*, which were all upregulated. ESCO2 is critical for the cohesion of sister chromatids during DNA replication and DNA damage repair [63]. Besides its role in DNA repair, *CBX1* has opposite effects in differentiated cells and pluripotent stem cells: it is required for the differentiation of differentiated cells while it maintains pluripotency in pluripotent stem cells [64]. Conversely, SUV39H1 regulates cell migration and tumor growth [65]. While *ESCO2* was also upregulated in R2 cells (although 2.5-fold less than in R1 cells), R2 cells highly downregulated the chromatin remodeling genes *HDAC4* and *DOT1L*. HDAC4 promotes growth of colon cancer cells via repression of p21 [66]. DOT1L deficiency leads to the dysregulation of cell cycle regulators and G1 cell cycle arrest [67].

Collectively, our findings suggested that R1 cells might have had a more resistant phenotype than R2 cells. This hypothesis was further supported by the finding that R2 cells lost the expression of *EIF4B* (Figure 3E), a substrate of the mTOR pathway, required for cell proliferation and survival of cancer cells [68]. Our analysis also identified *EGF* and *MYCN* as two de novo expressed genes in the population of resistant cells (Figure 3E). EGF has been reported to induce MYCN expression and to favor tumor growth [69].

Overexpression of CD24 and Muc1 (CD227) in R1 cells was confirmed using flow cytometry (Figure 3F). Moreover, we confirmed unaltered and decreased mTOR activity in R1 and R2 cells, respectively, by staining for the downstream mTOR target S6RP. We found that most of the phospho-S6RP (pS6RP-S235/236 and pS6RP-S240/244) expression localized at the cell colonies’ edges (Figure 3G). Mathematical models suggest that the growth in size of a cellular colony is due to a ring of growing and proliferating cells at the colony edge [70]. The mTOR signaling plays a major role in promoting cell-cycle progression, and downregulation of phospho-S6RP is a feature of slow-cycling cancer cells [71]. Thus, we hypothesize that parental and R1 cell-derived colonies contained proliferating cells at their edges expressing pS6RP. Proliferation is reduced in R2 cells, and this might be an effect of chemotherapy-induced mTOR pathway downregulation. Importantly, 5-FU chemoresistance in R1 cells was maintained over time, as indicated by high 5-FU IC50 value two months after their establishment (Supplementary Figure S2).

### 2.3. Differential Histology, Gene Expression Profiles, and Biological Functions of 5-FU Resistant Tu42 CRC Stem-Like Cells as Compared to Parental Cells In Vitro and In Vivo

**Figure 4:**
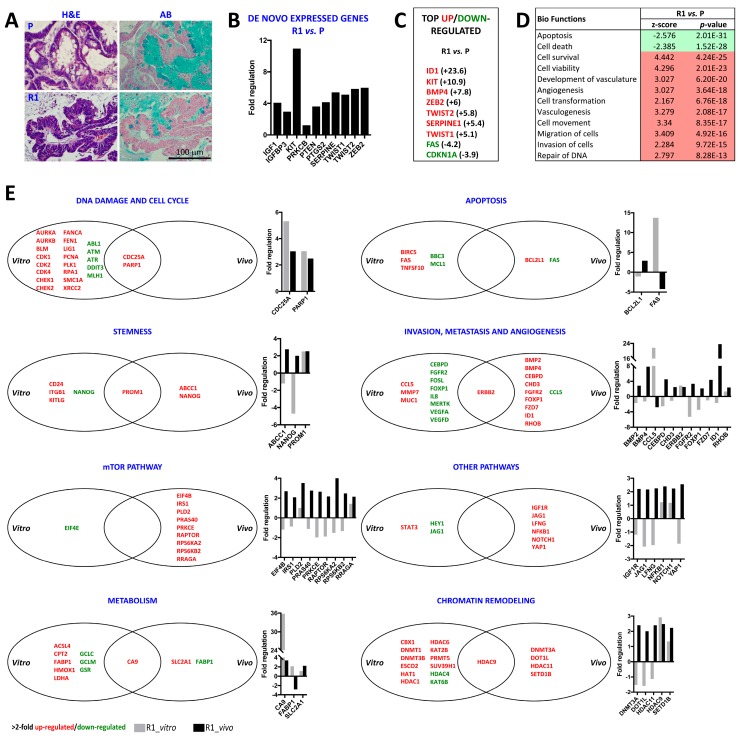
Differential histology, gene expression profiles and biological functions in R1 cells versus parental cells in vitro and in vivo. (**A**) PFA-fixed, paraffin-embedded serial sections of parental or R1 cell-derived tumor xenografts stained with hematoxylin and eosin (H&E) or alcian blue (AB). Characteristics of R1 cell-derived tumor xenograft include: a pseudostratified glandular epithelium with marked nuclear atypia and cellular overlapping and few mucinous goblet cells. Parental cell-derived tumor xenograft shows a lesser degree of architectural abnormalities including glands with low-grade cytologic dysplasia and abundant goblet cells. Pictures are representative of five histological sections/tumor. (**B**) Graph shows fold regulation (positive fold changes) of de novo expressed genes in R1 cell- versus parental cell-derived tumor xenograft. (**C**) List of the top up- or downregulated genes in R1 cell- versus parental cell-derived tumor xenograft obtained by real-time PCR arrays. (**D**) Biological functions ranked by *p*-value (from smallest or strongest to largest or weakest) predicted by IPA to be activated (red) or inhibited (green) in R1 cell- versus parental cell-derived tumor xenograft. (**E**) Venn diagrams show the lists of diverging and overlapping genes (for each of the following categories: DNA damage and cell cycle; apoptosis; stemness; invasion, metastasis and angiogenesis; mTOR pathway; other pathways; metabolism and chromatin remodeling) whose expression was altered (≥2.0 or ≤−2.0) in R1 cells as compared to parental cells in vitro and in vivo. Upregulated genes are in red, downregulated genes are in green. Graphs on the right of each Venn diagram show differential regulation of selected genes in vitro (grey bars) and in vivo (black bars).

We next established tumor xenografts using both parental and R1 cells. Morphologically, the tumors were different (Figure 4A).

Characteristics of R1 cell-derived tumor xenograft included a pseudostratified glandular epithelium with marked nuclear atypia and cellular overlapping, and few mucinous goblet cells. Conversely, parental cell-derived tumor xenograft showed a lesser degree of architectural abnormalities including glands with low-grade cytologic dysplasia and abundant goblet cells (Figure 4A). We therefore ran the same PCR arrays as before in the two tumor xenografts and compared their expression profiles. Table 2 lists the official full names for each gene found to be differentially regulated.

While we could not detect any mRNA transcript for *IGF1*, *IGFBP3*, *KIT*, *PRKCB*, *PTEN*, *PTGS2*, *SERPINE*, *TWIST1*, *TWIST2*, and *ZEB2* in vitro, all these genes were expressed in vivo (Figure 4B), possibly indicating different promoters’ methylation status or different microRNA regulation in the two conditions. Importantly, all the above-mentioned genes were upregulated in R1 cell-derived tumor xenograft with respect to parental cell-derived tumor xenograft (Figure 4B) and some of them (*KIT*, *SERPINE*, *TWIST1*, *TWIST*2, and *ZEB2*) could even be included in the top upregulated gene list of R1 cell-derived tumor xenograft versus parental cell-derived tumor xenograft (Figure 4C). These genes encode for proteins with well-known roles in cancer progression and normal stem cell maintenance (*KIT* [72]), angiogenesis (*SERPINE* [73]), and epithelial-mesenchymal transition (*TWIST1*, *TWIST2*, and *ZEB2* [74,75]). The list of top upregulated genes in R1 cell-derived tumor xenograft versus parental cell-derived tumor xenograft also included *ID1* and *BMP4* (Figure 4C). Moreover, *FAS* and *CDKN1A* were identified as the top downregulated genes in R1 cell-derived tumor xenograft versus parental cell-derived tumor xenograft (Figure 4C). Biological functions associated with cancer were predicted by IPA to be activated in R1 cell-derived tumor xenograft versus parental cell-derived tumor xenograft, while biological functions associated with death were predicted to be inactivated (Figure 4D).

Interestingly, we found that only a few of the genes which were upregulated in vitro by R1 cells were also upregulated in vivo. These included *CDC25A*, *PARP1*, *PROM1*, *ERBB2*, *CA9*, and *HDAC9* (Figure 4E). None of the genes which were downregulated in vitro were also downregulated in vivo. Opposing trends were observed for *FAS*, *NANOG*, *CCL5*, *CEBPD*, *FGFR2*, *FOXP1*, *JAG1*, and *FABP1*. As expected, in vivo, fewer of the genes associated with DNA damage and cell cycle, metabolism, or chromatin remodeling were changed. The greatest changes affected the invasion, metastasis and angiogenesis gene category, with *ID1* and *BMP4* being the most two upregulated genes. Furthermore, many genes in the mTOR pathway category were upregulated in vivo by R1 cells. Most of these genes (*EIF4B*, *PRAS40*, *RAPTOR*, *RPS6KA2*, *RPS6KB2*, and *RRAGA*) are directly or indirectly involved in the mTORC1 pathway [50]. This agrees with our previous observation that mTORC1 pathway activation is a feature of colorectal tumors [76].

### 2.4. ID1-Expressing Cell Enrichment Is a Feature of 5-FU Resistant Tu42 Cell-Derived Tumor Xenograft

**Figure 5:**
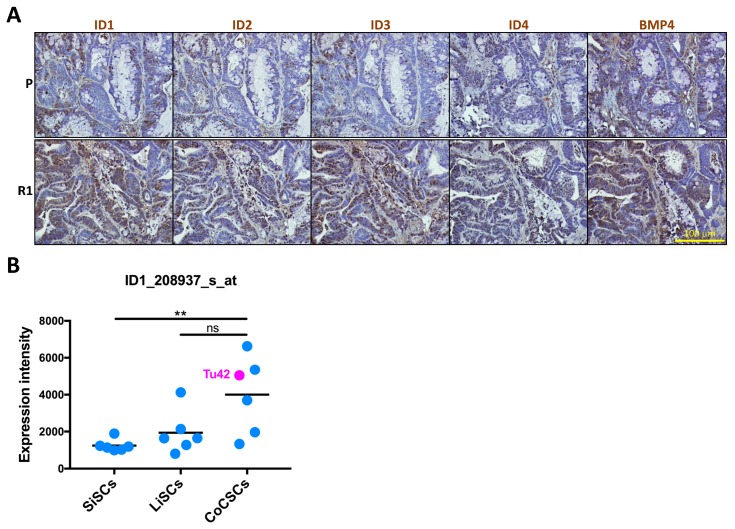
ID1-expressing cell enrichment is a feature of 5-FU resistant Tu42 cell-derived tumor xenograft. (**A**) PFA-fixed, paraffin-embedded serial sections of parental or R1 cell-derived tumor xenografts stained with ID1, ID2, ID3, ID4, or BMP4 antibodies (DAB, brown color). Pictures are representative of five histological stains per tumor. (**B**) Dot plot with line in the mean showing *ID1* expression obtained from quantile normalized microarray datasets of short-term feeder-expanded normal small or large intestinal stem cells (SiSCs and LiSCs, respectively) and CRC stem cells (CoCSCs). Each dot represents one sample. (ns. *p* > 0.05; ** *p* ≤ 0.01).

This study has identified *ID1* as the top upregulated gene in R1 cell-derived tumor xenograft. Using immunohistochemistry, we confirmed ID1 overexpression at the protein level (Figure 5A).

Interestingly, both ID1 positive and negative cells could be observed, leading to the speculation that ID1 could mark a subpopulation of cells with stemness features in the tumor. To establish ID1 as a stemness marker, the presence of ID1 in normal intestinal stem cells and its overexpression in cancerous intestinal stem cells need first to be demonstrated. To this purpose, we retrospectively compared the levels of *ID1* in short-term feeder-expanded primary epithelial cells isolated from human fetal small or large intestine to short-term feeder-expanded primary epithelial cells isolated from CRC biopsies. All three-cell types were cultured under conditions promoting the enrichment of cells with stemness features and are herein indicated as SiSCs, LiSCs, and CoCSCs, respectively [13,77].

**Figure 6:**
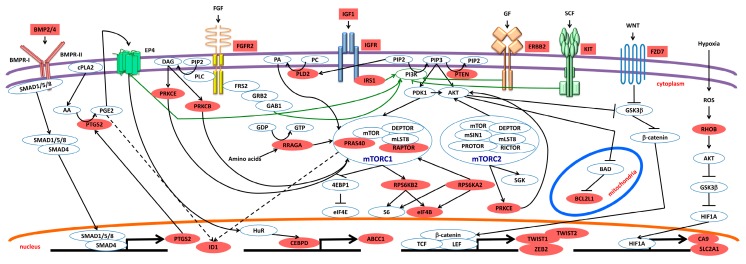
Potential activated pathways and targets in R1-cell derived tumor xenograft. Solid arrows indicate connections that are well established. Dashed arrows indicate less well established and/or more controversial. Light red color denotes detected upregulation.

We previously found intrinsic differences between SiSCs and LiSCs, with a tendency for cells expanded from the large intestine to be more closely related to cancer than their small intestine counterparts, possibly explaining why cancer is 20 times more prevalent in the large intestine than the small intestine in humans [77]. Accordingly, while *ID1* levels were statistically significant lower in SiSCs with respect to CoCSCs, there was no significant difference between LiSCs and CoCSCs, although *ID1* levels tended to be higher in CoCSCs (Figure 5B). These observations seem to suggest that *ID1*/ID1 could indeed mark cells with stemness features in our system. *ID1*/ID1 might be enriched in the cell-of-origin of intestinal cancer besides being expressed by cancer-propagating cells possessing stemness properties. Not only ID1, but also additional ID family members might identify cells with these characteristics. Indeed, we found ID2-4 to also be expressed (Figure 5A). While ID1 was detected in both cytoplasm and nucleus, ID2 and ID3 showed a clear nuclear expression, again confined to a subset of cells. Conversely, ID4 was weakly expressed (Figure 5A). IDs have been described as BMP4 targets in different cell types including embryonic stem cells [78]. In agreement with real-time PCR arrays, BMP4 was highly expressed in tumor sections (Figure 5A). Potential activated pathways and targets in R1-cell derived tumor xenograft are shown in Figure 6.

## 3. Discussion

Chemotherapeutic resistance represents a major treatment obstacle as most cancer patients ultimately relapse, becoming refractory to additional chemotherapeutic drugs.

Panels of chemotherapy resistant cancer cell lines have been generated in order to establish phenotypic signatures that might help predict chemotherapeutic sensitivity. However, despite the number of reported studies, there is no standardized protocol for reproducible generation of chemotherapeutic resistant cells. Both pulse and incremental methods have been described. However, a comparison of gene expression in the respective treatment-derivatives remains incomplete. Moreover, to our knowledge, re-transplantation into an in vivo setting of such in vitro-established resistant cell lines has been poorly studied.

In this study, we aimed at generating 5-FU resistant cells lines from five patient-derived CRC stem cell lines previously isolated and characterized in our lab [13]. Our data suggested that *PTEN*/*MMAC1*/*TEP1* status could be a key determinant for successful generation of drug resistant cells. Indeed, only Tu42 cells, which downregulated *PTEN*/*MMAC1*/*TEP1*, could survive long-term 5-FU treatment. *PTEN*/*MMAC1*/*TEP1* is the most frequently inactivated tumor suppressor gene in sporadic cancer [79] and acts by inhibiting cancer cell proliferation and invasiveness and promoting apoptosis through its antagonism of PI3K. Tu42 cells did not express *PTEN*/*MMAC1*/*TEP1* in vitro; however, *PTEN*/*MMAC1*/*TEP1* expression could be detected in Tu42 cell-derived tumor xenograft, indicating reversible *PTEN*/*MMAC1*/*TEP1* loss in these cells.

We used two treatment regimens to generate resistant cells, and we compared the transcriptional profiles of the resistant cell derivatives by focusing on almost 600 genes with roles in cell cycle, DNA repair, apoptosis, stemness, metastasis, metabolism, and epigenetics. We identified similarities and differences of 5-FU resistant cells generated by intermittent treatment with the IC50 drug dose or continuous treatment with escalating drug doses. Similarities included upregulation of *CA9*, *MUC1*, and *CD24*, and downregulation of *HEY1*, *NANOG*, and *IL8*. Similarities also included de novo acquisition of two potent oncogenes with crucial roles in cell growth and proliferation, *EGF* and *MYC* [80]. Despite these similarities, 5-FU resistant cells generated by continuous treatment with escalating drug doses appeared to be less resistant than the other set of treated cells, showing downregulation of numerous genes involved in invasion, metastasis, and angiogenesis, as well as mTOR pathway downregulation. From a therapeutic standpoint, this data suggests that escalating the dose of chemotherapy might enhance efficiency. However, at the beginning of the treatment, cells that are being treated with escalating drug doses might pose a threat. Indeed, our data of TRA-2-49 upregulation together with EpCAM, Muc2, and Villin downregulation in 20 μM 5-FU resistant cells seems to suggest mechanisms of epithelial-mesenchymal transition, oncogenic dedifferentiation, and acquisition of stemness features [81,82]. It is worth spending a few words on EpCAM because it might help track phenotypic changes during chemotherapeutic treatment: EpCAM-expressing cells proliferated in response to 10 μM 5-FU; later doubling of the 5-FU concentration resulted in the downregulation of EpCAM, however EpCAM expression could be restored when the cells were cultured in 3D matrices, which represents an assay of cell differentiation. This data highlights the dynamic nature of CRC stem-like cells which undergo constant dynamic changes to adapt and survive in adverse conditions.

By engrafting 5-FU resistant cells generated by intermittent treatment with the IC50 drug dose in the mouse host, we confirmed the capacity of such cells to regenerate a tumor in vivo. The observation that several genes implicated in stem cell maintenance and cancer progression were de novo expressed, in vivo indicated that resistant cells continued to adapt in the new environment, even in the absence of further chemotherapeutic insult.

Interestingly, while resistant cells generated by intermittent treatment with the IC50 5-FU dose and control parental cells passaged in parallel did not differ for the expression of *ID1* in vitro, *ID1* was the top upregulated gene in the resistant cell-derived tumor xenograft as compared to the control tumor xenograft. Using immunohistochemistry, we confirmed ID1 overexpression in the resistant cell-derived tumor xenograft at the protein level. Both ID1 positive and negative cells could be observed. The mechanisms driving this intratumoral heterogeneity remain to be fully investigated. A tumor is a complex ecosystem of relatively differentiated cancer cells and CSCs as well as other cell types. In the tumor, individual cells display a diverse set of characteristics and function together to support the growth and maintenance of the tumor as a whole. ID1-expressing cells within the drug resistant cancer cell pool might have preferentially proliferated following transplantation into the mouse host, while the ID1-expressing cells within the untreated cancer cell pool might have had a decreased proliferation potential. Emerging technologies including single-cell transcriptomics could allow us to test our hypothesis and determine whether ID1 is restricted to a CSC subpopulation in the primary colorectal tumor and is pervasively expressed in secondary lesions emerging after completion of chemotherapy. Undoubtedly, our retrospective analysis of microarray *ID1*-expression indicated that *ID1* is already expressed in normal intestinal stem cells in accordance with a previous study [83], and therefore, the *ID1*-expressing population might play a role in tumor initiation besides propagation.

In conclusion, our study connects the capacity for adaptation and propagation of CRC stem-like cells after chemotherapeutic pressures, knowledge that could be exploited in future therapeutic strategies.

## 4. Materials and Methods

### 4.1. Specimens

Tumor tissues were obtained by David Geller (surgeon, University of Pittsburgh Medical Center, Pittsburgh, PA, USA) after informed consent in accordance with institutional review board (IRB) protocols from patients with colorectal adenocarcinoma (IRB:PRO08010372).

### 4.2. Cell Culture and Determination of 5-FU IC50

Tu11, Tu14, Tu27, Tu28, and Tu42 CRC-stem like cells were cultured in Dulbecco’s Modified Eagle Medium:Nutrient Mixture F-12 (DMEM/F12, Corning, cat. no. 10090CV; Fisher Scientific, Pittsburgh, PA, USA) containing 1% insulin-transferrin-selenium (ITS, Corning, cat. no. 25800CR) and 0.5% fetal bovine serum (FBS, Atlanta Biologicals, cat. no. S1245OH; Fisher Scientific). To establish 5-FU IC50, cells were seeded onto 96-well plates (10,000 cells/well) in 100 µL of culture medium overnight, and then treated in triplicate with serial dilutions of 5-FU (10–250 µM) (Sigma-Aldrich, cat. no. F6627-1G; Saint Louis, MO, USA). The number of viable cells was measured using the MTS-based CellTiter 96 AQueous Assay (Promega, cat. no. G3582; Madison, WI, USA) 72 h after drug exposure. 20 µL of the MTS reagent were added to each well, and the absorbance was recorded at 490 nm after 4 h incubation at 37 °C. Data were expressed as mean percentage (±SD) of cell numbers relative to control culture.

### 4.3. Cytotoxicity Assay

Tu27, Tu28, and Tu42 cells were seeded onto 96-well plates (10,000 cells/well) in 100 µL of culture medium overnight, and then treated in triplicate with serial dilutions of 5-FU (10–500 µM) for 72 h. The number of viable cells was measured as above described.

### 4.4. Establishment of 5-FU Resistant Cell Lines

Tu42 cells were treated with 100 µM 5-FU for 72 h. Surviving cells were allowed to recover 21 weeks in fresh media before a second pulse of the drug was given. Surviving cells were allowed to recover additional 14 weeks before further applications (R1 cells). Alternatively, 5-FU resistant cell lines (R2 cells) were established after six sequential treatments with 5-FU during an 8-month period (5 µM and 100 µM, initial and final 5-FU concentration, respectively). Control parental cells were passaged in parallel.

### 4.5. Organoid Preparation

Tu42 cells were suspended in 50μL Growth Factor Reduced (GFR) Matrigel (Corning, cat.no. 354230) and plated in tissue culture dishes at low density (50,000 cells/well of 48-well plate). After solidification at 37°C for 30 min, the Matrigel was overlaid with culture media. Whole intact Tu42 organoids were fixed 30 min in 4% PFA, and embedded in optimal cutting temperature compound (OCT, Fisher Scientific, cat. no. 4853) for cryostat sectioning.

### 4.6. Immunostains

For immunofluorescence, tumor cells were detached using Trypsin/EDTA (Corning, cat. no. 25053CI). After rinsing in phosphate-buffered saline (PBS, Corning, cat. no. 21040CV), drops of the cell suspension were directly placed onto microscope slides, air-dried, and fixed in cold acetone for 10 min. Alternatively, cells were grown on Nunc Lab Tek 4-well chamber slides (Fisher Scientific, cat. no. 177437), air-dried, and fixed as above. Fixed slides containing cells or 5-micron-thick frozen sections of organoids were washed with PBS, blocked with 3% bovine serum albumin (BSA, Fisher Scientific, cat. no. 9048468) in 0.05% Tween 20-containing PBS (PBST) for 30 min, before incubation for 1 h with antibodies against: Wheat germ agglutinin/Alexa Fluor 594 (Molecular Probes, cat.no. W11262; Eugene, OR, USA), Lamin B1 (Abcam, cat. no. ab16048; Cambridge, MA, USA), SF2 (Abcam, cat. no. ab38017), Fibrillarin (Abcam, cat. no. ab4566), Nucleostemin (Abcam, cat. no. ab70346), Nucleophosmin (Abcam, cat. no. ab10530), PML (Abcam, cat. no. ab53773), Ki-67 (Abcam, cat. no. ab15580), TRA-2-49 (Abcam, cat. no. ab17973), EpCAM (Abcam, cat. no. ab32392), Muc2 (Santa Cruz, cat. no. sc-7314; Santa Cruz, CA, USA), Villin (AbD Serotec, cat. no. MAB1671; Raleigh, NC, USA), phospho-S6RP Ser235/236 (Cell Signaling Technology, cat. no. 4858; Boston, MA, USA) or phospho-S6RP Ser240/244 (Cell Signaling Technology, cat. no. 5364). After primary antibody incubation, slides were washed three times with PBST and incubated, when necessary, with Alexa Fluor 488 and/or 594 conjugated secondary antibody (Invitrogen; Carlsbad, CA, USA) for 30 min. Slides were washed as previously described, and coverslips mounted onto slides using Hank’s Salt (HBSS, HyClone, cat. no. SH3026801; Fisher Scientific) containing 50% glycerol (Fisher Scientific, cat. no. BP2291) and 10 µg/mL Hoechst 33342 (Molecular Probes, cat. no. H21492). Images were obtained using the Olympus IX71 inverted fluorescence microscope (Olympus America, Center Valley, PA, USA).

For histological stains, 5mm tissues were fixed in 4% paraformaldehyde (PFA) overnight at 4 °C and then stored in 70% ethanol until paraffin embedding. Paraffin blocks were cut into 7-micron-thick sections. Hematoxylin and eosin and Alcian blue stains were performed as described elsewhere. For immunohistochemistry, paraffin sections were deparaffinized in xylene and rehydrated in graded alcohol. Endogenous peroxidase was blocked using 3% hydrogen peroxide for 10 min. Antigen unmasking was performed using 10 mM sodium citrate buffer pH 6.0. Reaction was carried out using R.T.U. VECTASTAIN Universal ABC Kit (cat. no. PK-7800; Vector Laboratories, Burlingame, CA, USA). Specifically, non-specific binding was blocked by incubating the slides in horse serum for 15 min. The slides were then exposed to primary antibodies against ID1 (LSBio, cat. no. LS-C165110; Seattle, WA, USA), ID2 (LSBio, cat. no. LS-C50475), ID3 (LSBio, cat. no. LS-B3365), ID4 (LSBio, cat. no. LS-B9923) or BMP4 (LSBio, Millipore, cat. no. ABD83) overnight at 4 °C, and later incubated with VECTASTAIN biotinylated pan-specific universal secondary antibody for 10 min, followed by VECTASTAIN streptavidin/peroxidase complex reagent for 5 min and ImmPACT DAB substrate incubation (Vector Laboratories, cat. no. SK-4105). Immunostained sections were counterstained with Gill’s hematoxylin, dehydrated with graded strengths of alcohols, cleared in xylene, and finally coverslipped using a Permount mounting media (Fisher Scientific, cat. no. SP15-100).

### 4.7. RNA Extraction, cDNA Synthesis, PCR Arrays

Total RNA was isolated from cells or tissues stored in RNAlater reagent (QIAGEN, cat. no. 76104; Valencia, CA, USA) using the RNeasy Mini kit (QIAGEN, cat. no. 74104), according to the manufacturer’s instructions. Potentially contaminating genomic DNA was digested using DNase (QIAGEN, cat. no. 79254). Purity of isolated RNA was determined by measuring ratio of the optical density of the samples at 260 and 280 nm. The OD_260_/_280_ ratio was ranging from 2.06 to 2.12 for all samples. cDNAs were synthesized using the RT^2^ First Strand Kit (QIAGEN, cat. no. 330401). QIAGEN’s RT² PCR Array Human RNA QC (cat. no. PAHS-999Z) was used to test the quality of the RNA samples before proceeding with the PCR arrays. A total of 588 genes were analyzed in duplicate using the following QIAGEN’s RT² Profiler PCR arrays: Human mTOR Signaling (cat. no. PAHS-098A), Human Cancer Stem Cells (cat. no. PAHS-176A), Human Signal Transduction Pathway Finder (cat. no. PAHS-014ZA), Human Damage Signaling Pathway (cat. no. PAHS-029ZA), Human Cancer Targets (cat. no. PAHS-507ZA), Human Epigenetic Chromatin Modification Enzymes (cat. no. PAHS-085ZA), and Human Epigenetic Chromatin Remodeling Factors (cat. no. PAHS-086ZA). 0.5 µg total RNA was used for each plate. A StepOnePlus™ Real-Time PCR System (Applied Biosystems, Foster City, CA, USA) was used for gene amplification. Dissociation (melting) curve analysis was performed to verify PCR specificity. The analysis was performed automatically according to the SABiosciences company web portal (QIAGEN), and further confirmed manually using the 2^−ΔΔ*Ct*^ method. Changes in gene expression obtained from two technical replicates were illustrated as fold regulations.

Levels of *PTEN/MMAC1*/*TEP1* (204054_at; 217492_s_at; 204053_x_at) in short-term feeder-expanded CRC stem cells (Tu11, Tu14, Tu27, Tu28, and Tu42) were retrieved from a microarray dataset previously obtained in the laboratory (unpublished data). Levels of *ID1* (208937_s_at) were retrieved from quantile normalized microarray datasets of short-term feeder-expanded normal intestinal stem cells [77] and CRC stem cells (Tu14, Tu18, Tu22, Tu25, Tu28, and Tu42) (unpublished data).

### 4.8. Ingenuity Pathway Analysis (IPA)

To identify biological functions perturbed in response to chemotherapy-induced selective pressure, the dataset representing the log2 (fold change) of the expression level of 439 genes analyzed under different culture conditions was imported into the QIAGEN’s IPA tool. Both core and comparison analyses were performed. The following settings were used: the Ingenuity Knowledge Base (genes only) database was used as a reference set; direct and indirect relationships were included, as well as molecule relationships with endogenous chemicals, with a maximum of 35 focus molecules per network and a maximum of 25 networks per analysis. All node types and data sources were included. The confidence level was set to include experimentally observed relationships. Only human genes were included, and genes were filtered for colon cancer cell lines. All mutations were included. A 2-fold cut-off on log2 (fold changes) was applied.

### 4.9. Flow Cytometry

Cells were incubated in the dark for 1 h with FITC Mouse Anti-Human CD24 (BD Pharmingen, cat. no. 555427; San Diego, CA, USA) or FITC Mouse Anti-Human CD227 (BD Pharmingen, cat. no. 559774). Cells were centrifuged, washed three times, and the final cell pellet was resuspended in 400 µL of flow buffer with Sytox Blue dye (Molecular Probes, cat.no. S34857). Cells were analyzed using a Miltenyi MACSQuant and FlowJo software (Tree Star, Inc; Ashland, OR, USA).

### 4.10. Animals

BALB/c nude mice were purchased from Charles River (Wilmington, MA, USA), and bred and housed in the Division of Laboratory Animal Resources (DLAR) facility at the University of Pittsburgh. Experimental protocols followed US National Institutes of Health (NIH) guidelines for animal care and were approved by the Institutional Animal Care and Use Committee (IACUC) at the University of Pittsburgh. Tumor cells (2.5 × 10^5^) were suspended in HBSS:Matrigel (1:1) and injected s.c. into both flanks of 5-week-old mice (*n* = 3). Xenografted tumors were excised, fixed in buffered formalin and embedded in paraffin for histological and immunohistochemical examination or stored in RNAlater for RNA isolation.

### 4.11. Statistical Analysis

*P*-values for IPA predictions were calculated using Fisher’s exact test. A biofunction was considered to be significantly activated or inhibited based on a z-score ≥2.0 or ≤−2.0, respectively and *p*-value ≤ 0.01. Expression of pS6RP-S235/236 and pS6RP-S240/244 in parental, R1, and R2 cell-derived colonies was compared using unpaired *t*-test with Welch’s correction. The expression intensities of *ID1* in normal intestinal stem cells and colorectal CSCs were compared using unpaired *t*-test.

## Figures and Tables

**Table 1:** Official full names for genes differentially expressed in vitro.

Official Symbol	Official Full Name
*ABCC1*	ATP binding cassette subfamily C member 1
*ABL1*	ABL proto-oncogene 1, non-receptor tyrosine kinase
*ACSL4*	Acyl-CoA synthetase long chain family member 4
*AKT2*	AKT serine/threonine kinase 2
*ATM*	ATM serine/threonine kinase
*ATR*	ATR serine/threonine kinase
*ATRX*	ATRX, chromatin remodeler
*AURKA*	Aurora kinase A
*AURKB*	Aurora kinase B
*BBC3*	BCL2 binding component 3
*BCL2L1*	BCL2 like 1, Bcl-xl
*BIRC5*	Baculoviral IAP repeat containing 5, Survivin
*BLM*	BLM RecQ like helicase
*BMP4*	Bone morphogenetic protein 4
*CA9*	Carbonic anhydrase 9
*CBX1*	Chromobox 1
*CCL5*	C-C motif chemokine ligand 5
*CD24*	CD24 molecule
*CDC25A*	Cell division cycle 25A
*CDC42*	Cell division cycle 42
*CDK1*	Cyclin dependent kinase 1
*CDK2*	Cyclin dependent kinase 2
*CDK4*	Cyclin dependent kinase 4
*CDK5*	Cyclin dependent kinase 5
*CEBPD*	CCAAT enhancer binding protein delta
*CHD3*	Chromodomain helicase DNA binding protein 3
*CHEK1*	Checkpoint kinase 1
*CHEK2*	Checkpoint kinase 2
*CPT2*	Carnitine palmitoyltransferase 2
*CTSB*	Cathepsin B
*CTSD*	Cathepsin D
*CTSL*	Cathepsin L
*DDIT3*	DNA-damage inducible transcript 3
*DNMT1*	DNA methyltransferase 1
*DNMT3A*	DNA methyltransferase 3 alpha
*DNMT3B*	DNA methyltransferase 3 beta
*DOT1L*	DOT1 like histone lysine methyltransferase
*EGF*	Epidermal growth factor
*EIF4B*	Eukaryotic translation initiation factor 4B
*EIF4E*	Eukaryotic translation initiation factor 4E
*ERBB2*	Erb-b2 receptor tyrosine kinase 2
*ESCO2*	Establishment of sister chromatid cohesion N-acetyltransferase 2
*FABP1*	Fatty acid binding protein 1
*FANCA*	FA complementation group A
*FANCG*	FA complementation group G
*FAS*	Fas (TNF receptor superfamily member 6)
*FEN1*	Flap structure-specific endonuclease 1
*FGFR2*	Fibroblast growth factor receptor 2
*FOSL1*	FOS like 1, AP-1 transcription factor subunit
*FOXP1*	Forkhead box P1
*FZD7*	Frizzled class receptor 7
*GADD45A*	Growth arrest and DNA damage inducible alpha
*GCLC*	Glutamate-cysteine ligase catalytic subunit
*GCLM*	Glutamate-cysteine ligase, modifier subunit
*GSR*	Glutathione-disulfide reductase
*HAT1*	Histone acetyltransferase 1
*HDAC1*	Histone deacetylase 1
*HDAC11*	Histone deacetylase 11
*HDAC4*	Histone deacetylase 4
*HDAC6*	Histone deacetylase 6
*HDAC7*	Histone deacetylase 7
*HDAC9*	Histone deacetylase 9
*HES1*	Hes family bHLH transcription factor 1
*HEY1*	Hairy/enhancer-of-split related with YRPW motif 1
*HMOX1*	Heme oxygenase 1
*ID1*	Inhibitor of DNA binding 1, HLH protein
*IL8*	Interleukin 8
*IRS1*	Insulin receptor substrate 1
*ITGB1*	Integrin Subunit Beta 1
*JAG1*	Jagged 1
*KAT2A*	Lysine acetyltransferase 2A
*KAT2B*	Lysine acetyltransferase 2B
*KAT6B*	Lysine acetyltransferase 6B
*KDM5C*	Lysine demethylase 5C
*KITLG*	KIT ligand
*LDHA*	Lactate dehydrogenase A
*LFNG*	LFNG O-fucosylpeptide 3-beta-N-acetylglucosaminyltransferase
*LIG1*	DNA ligase 1
*MAML1*	Mastermind like transcriptional coactivator 1
*MCL1*	MCL1, BCL2 family apoptosis regulator
*MERTK*	MER proto-oncogene, tyrosine kinase
*MLH1*	MutL homolog 1
*MMP7*	Matrix metallopeptidase 7
*MUC1*	Mucin 1
*MYCN*	MYCN proto-oncogene, bHLH transcription factor
*NANOG*	Nanog
*NCOA1*	Nuclear receptor coactivator 1
*NOTCH2*	Notch 2
*NQO1*	NAD(P)H quinone dehydrogenase 1
*PARP1*	Poly(ADP-ribose) polymerase 1
*PCNA*	Proliferating cell nuclear antigen
*PLAUR*	Plasminogen activator, urokinase receptor
*PLD2*	Phospholipase D2
*PLK1*	Polo like kinase 1
*PPP2R4*	Phosphotyrosyl phosphatase activator
*PRAS40*	Proline-rich Akt substrate 40 kDa
*PRKCE*	Protein kinase C epsilon
*PRMT5*	Protein arginine methyltransferase 5
*PROM1*	Prominin 1, CD133
*RAPTOR*	Regulatory associated protein of mTOR
*RHOB*	Ras homolog family member B
*RPA1*	Replication protein A1
*RPS6KA2*	Ribosomal protein S6 kinase A2
*RPS6KB2*	Ribosomal protein S6 kinase B2
*SETD1A*	SET domain containing 1A
*SETD1B*	SET domain containing 1B
*SMC1A*	Structural maintenance of chromosomes 1A
*STAT3*	Signal transducer and activator of transcription 3
*SUV39H1*	Suppressor of variegation 3-9 homolog 1
*TGFBR1*	Transforming growth factor beta receptor 1
*TNFSF10*	TNF superfamily member 10, TRAIL
*VEGFA*	Vascular endothelial growth factor A
*VEGFD*	Vascular endothelial growth factor D
*XRCC2*	X-ray repair cross complementing 2

**Table 2:** Official full names for genes differentially expressed in vivo.

Official Symbol	Official Full Name
*ABCC1*	ATP binding cassette subfamily C member 1
*BCL2L1*	BCL2 like 1, Bcl-xl
*BMP2*	Bone morphogenetic protein 2
*BMP4*	Bone morphogenetic protein 4
*CA9*	Carbonic anhydrase 9
*CEBPD*	CCAAT enhancer binding protein delta
*CDC25A*	Cell division cycle 25A
*CHD3*	Chromodomain helicase DNA binding protein 3
*DNMT3A*	DNA methyltransferase 3 alpha
*DOT1L*	DOT1 like histone lysine methyltransferase
*ERBB2*	Erb-b2 receptor tyrosine kinase 2
*EIF4B*	Eukaryotic translation initiation factor 4B
*FAS*	Fas (TNF receptor superfamily member 6)
*FABP1*	Fatty acid binding protein 1
*FGFR2*	Fibroblast growth factor receptor 2
*FOXP1*	Forkhead box P1
*FZD7*	Frizzled class receptor 7
*HDAC11*	Histone deacetylase 11
*HDAC9*	Histone deacetylase 9
*ID1*	Inhibitor of DNA binding 1, HLH protein
*IGF1*	Insulin like growth factor 1
*IGF1R*	Insulin like growth factor 1 receptor
*IGFBP3*	insulin like growth factor binding protein 3
*IRS1*	Insulin receptor substrate 1
*JAG1*	Jagged 1
*KIT*	KIT proto-oncogene receptor tyrosine kinase
*LFNG*	LFNG O-fucosylpeptide 3-beta-N-acetylglucosaminyltransferase
*NANOG*	Nanog
*NOTCH1*	Notch 1
*NFKB1*	Nuclear factor kappa B subunit 1
*PTEN*	Phosphatase and tensin homolog
*PLD2*	Phospholipase D2
*PARP1*	Poly(ADP-ribose) polymerase 1
*PRAS40*	Proline-rich Akt substrate 40 kDa
*PROM1*	Prominin 1, CD133
*PTGS2*	Prostaglandin-endoperoxide synthase 2
*PRKCB*	Protein kinase C beta
*PRKCE*	Protein kinase C epsilon
*RHOB*	Ras homolog family member B
*RRAGA*	Ras related GTP binding A
*RAPTOR*	Regulatory associated protein of mTOR
*RPS6KA2*	Ribosomal protein S6 kinase A2
*RPS6KB2*	Ribosomal protein S6 kinase B2
*SERPINE1*	Serpin family E member 1
*SETD1B*	SET domain containing 1B
*SLC2A1*	Solute carrier family 2 member 1, GLUT-1
*TWIST1*	Twist family bHLH transcription factor 1
*TWIST2*	Twist family bHLH transcription factor 2
*YAP1*	Yes associated protein 1
*ZEB2*	Zinc finger E-box binding homeobox 2

## Supplementary Materials

Supplementary materials can be found at https://www.mdpi.com/1422-0067/20/8/1817/s1.
