# Supplementary material for: Establishment and Characterization of 5-Fluorouracil-Resistant Human Colorectal Cancer Stem-Like Cells: Tumor Dynamics under Selection Pressure
Source: Int J Mol Sci. 2019 Apr 12;20(8):1817. doi: 10.3390/ijms20081817 (PMC6515384; doi:10.3390/ijms20081817)
Supplement: Supplementary file 1 [file ijms-20-01817-s001.pdf]

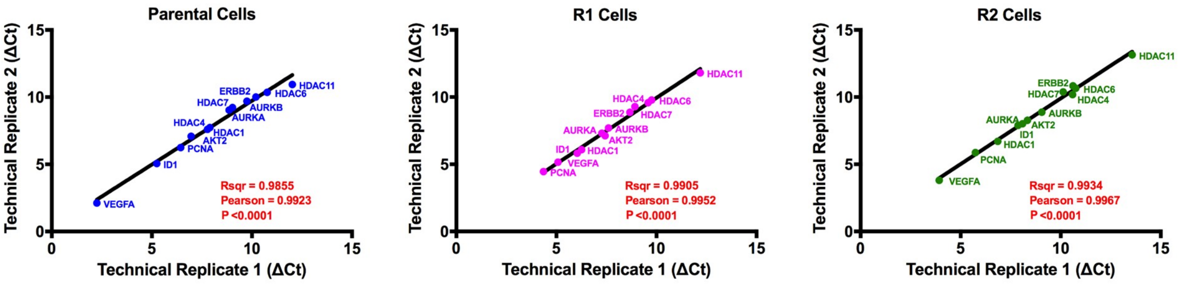

Supplementary Figure S1. Graphs of linear regression and Pearson correlation obtained from the ΔCt values of a dozen of randomly selected genes in two technical replicates of parental, R1 or R2 cells.

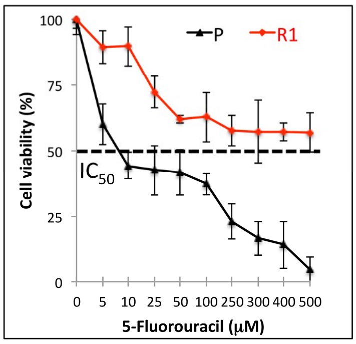

Supplementary Figure 2. Line graph shows cell viability (%) of parental (black line) or R1 (red line) cells (two months after their establishment) following treatment with vehicle or serial dilutions (10-500 μM) of 5-FU. Data are expressed as mean percentage (± SD) of cell numbers relative to control culture.
